# Long working hours, sleep-related problems, and near-misses/injuries in industrial settings using a nationally representative sample of workers in Japan

**DOI:** 10.1371/journal.pone.0219657

**Published:** 2019-07-15

**Authors:** Takashi Yamauchi, Takeshi Sasaki, Kunihiko Takahashi, Shigeo Umezaki, Masaya Takahashi, Toru Yoshikawa, Machi Suka, Hiroyuki Yanagisawa

**Affiliations:** 1 Department of Public Health and Environmental Medicine, The Jikei University School of Medicine, Tokyo, Japan; 2 Research Center for Overwork-Related Disorders, National Institute of Occupational Safety and Health, Kawasaki, Japan; 3 Department of Biostatistics, Nagoya University Graduate School of Medicine, Nagoya, Japan; 4 National Institute of Occupational Safety and Health, Kiyose, Japan; Universitat de Valencia, SPAIN

## Abstract

Long working hours and a lack of sleep have been suggested to negatively affect the safety of workers. Here, we examined the association between long working hours/sleep-related problems and near-misses/injuries in industrial settings using a nationally representative sample of workers in Japan. Based on the composition ratio of workers by industry, sex, and age in Japan, data from a web-based cross-sectional survey for 18,682 participant full-time workers (7,098 female and 11,584 male; mean age, 43.7 [standard deviation 11.1] years) were analyzed. Nearly 30% and 5% of participants reported any types of near-misses during the past six months and injuries during the past year, respectively. For all types of near-misses and some types of injuries, a significant difference in frequency distribution was observed by industry. After adjustment for demographic, job-, and life-related variables, participants who worked long hours (i.e., more than 51 hours per week) were more likely to report job-related near-misses/injuries than those who worked 35–40 hours per week. The presence of sleep-related problems was also significantly related to near-misses and injuries. However, while sleep-related problems were significantly associated with near-misses/injuries in all industries, the association between long working hours and near-misses/injuries differed by industry. Odds ratios for near-misses/injuries were strongly significant in the “transport/postal services” industry for those who worked more than 51 hours per week compared to those who worked 35–40 hours per week. Comprehensive protective measures for workers, including (1) reducing total hours of service/job-related fatigue, (2) maintaining sufficient sleep hours/good sleep, and (3) increasing awareness about the impact of overwork/long working hours and sleep-related problems on workers’ safety among employers, workers, clients/customers, and the general public might be effective for preventing near-misses and injuries in industrial settings among workers, especially those who work long hours in the “transport/postal services” industry.

## Introduction

Despite substantial prevention efforts, occupational injuries are a major problem worldwide,[1] including Japan.[2] Previous systematic reviews on occupational safety have suggested that fatigue due to long working hours, heavy workloads, and insufficient sleep/sleepiness have detrimental effects on job performance, decision-making, and, subsequently, safety.[3–7]

Previous epidemiological studies using a nationally representative sample of workers on the association between long working hours/sleep-related problems and injuries in industrial settings, adjusting for the effect of other factors associated with the risk of injuries (e.g., depression), have been conducted in western countries.[8–10] However, to our knowledge, no such study has been conducted in Asian countries where long working hours are more prevalent than in developed western countries.[11] In addition, these previous studies using nationally representative data focused only on occupational injuries and did not investigate “near-misses” for workers in specific industries. Recent reports have suggested that near-misses (i.e., “an unplanned event that did not result in injury, illness, or damage but had the potential to do so” [12]) and injuries, including severe injuries and fatalities, have similar causal factors.[13] As argued by previous reports,[14] from a preventive standpoint, viewing near-misses and injuries as having a common underlying cause may be effective, because major injuries are considered to be preceded by several near-misses.

Previous studies suggest that the type of near-misses/injuries and the impact of fatigue due to long working hours and sleep-related problems on these events differed by industry, including transport/postal services and medical/health/welfare industries.[7, 15, 16] Thus, the present study aimed to examine the association between long working hours and sleep-related problems and near-misses and injuries in industrial settings using a nationally representative sample of workers in Japan, where the proportion of workers who work 49 hours or more per week was higher than that in developed western countries.[11] A better understanding of the association between long working hours/sleep-related problems and near-misses/injuries by industry using nationally representative data of workers may contribute to the development of a national policy to promote preventive measures against occupational injuries. After adjusting for sociodemographic and job-related variables, we found that long working hours and sleep-related problems were associated with near-misses and injuries; however, the association differed by industry.

## Materials and methods

### Data source and study design

To investigate working and living conditions and health/safety-related outcomes among Japanese workers, a web-based cross-sectional survey of 30,000 workers was conducted in collaboration with a research company in Tokyo, Japan. This company has one of the largest online research panels in Japan with over 1.8 million voluntarily registered panelists (http://global.cross-m.co.jp/solution/online/index.html). To minimize selection bias, this survey selected a sample of Japanese workers aged 20 to 64 years based on the composition ratio of workers by industry, sex, and age in the Labour Force Survey by the Japanese Ministry of Internal Affairs and Communications.[17] The research company randomly sent an e-mail invitation for participation in the study to registered workers. Workers who provided web-based informed consent were selected to participate in the survey; they answered a self-rated questionnaire on the Internet which consisted of questions regarding demographic, job-related, and life-related variables as well as health/safety-related outcomes. The research company recruited participants until the total number of participants who completed the web-based questionnaire reached 30,000. The sample size for the present study was determined based on previous studies that reported the incidence of occupational injuries.[18]

The industry was categorized using the “Japan Standard Industrial Classification” established by the Japanese Ministry of Internal Affairs and Communications.[19] The proportions of potential participants by industry, sex, and age group in the present study were almost identical to that of labor force populations in the 2017 Labour Force Survey in Japan (S1 Appendix). In the present study, we specifically focused on the participants who were working in “construction,” “manufacturing,” “transport/postal services,” “wholesale/retail,” or “medical/health/welfare” industries. These five industries had the highest number of cases involving compensation for occupational injuries in Japan in 2017, accounting for 75% of compensated occupational injuries in all industries.[2]

**Fig 1:**
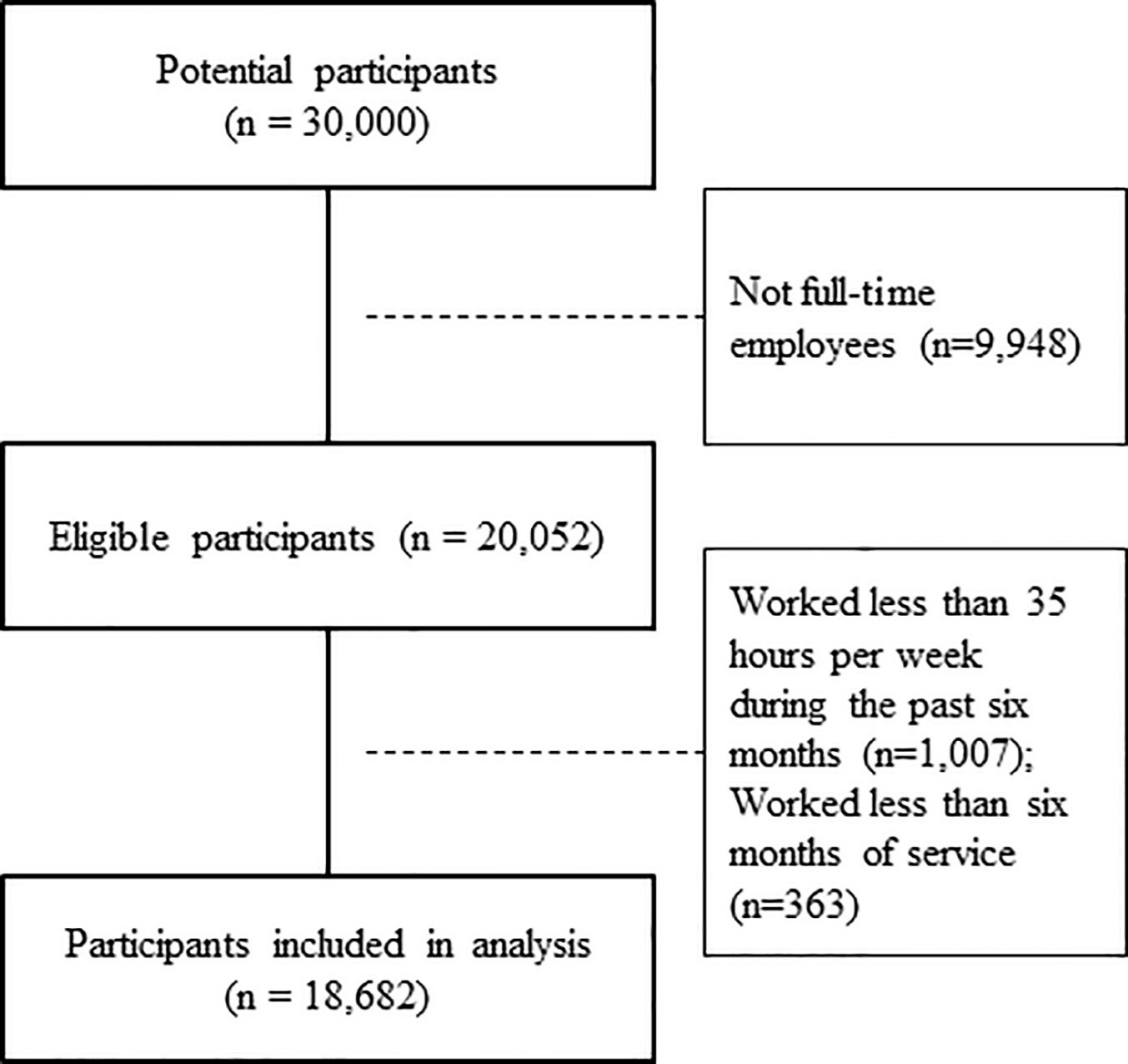
Participant selection.

To equalize working conditions among the participants, we excluded those who were not full-time employees; 20,052 participants remained eligible for the present study (Fig 1). Subsequently, we excluded participants who worked less than 35 hours per week or six months of service. No participant had missing data regarding the variables used for statistical analyses in the study.

The study protocol was approved by the institutional review board of the Jikei University School of Medicine, Tokyo, Japan in 2018 (No. 30-153(9174)).

### Measurements

The outcome measures of the present study were occupational injuries and near-misses. According to previous reports on near-misses,[12] we defined a near-miss as “an unplanned event that did not result in injury, illness, or damage but had the potential to do so.”

First, participants were asked whether they had experienced any near-misses in industrial settings during the past six months, under the instruction of the definition of a near-miss (“*Have you experienced any near-misses in industrial settings during the past six months*?”). Participants who answered “yes” were subsequently asked to select all types of near-misses they have experienced from the following five categories: (1) falling, (2) crashing/crashed, (3) getting caught in/between, getting cut, (4) traffic accident, and (5) others (e.g., burn, explosion). These categories were classified based on previous reports on near-misses.[2, 14]

Second, participants were asked whether they had had any occupational injuries during the past year (“*Have you experienced any occupational injuries during the past year*?”). Participants who answered “yes” were subsequently asked to select all types of injuries they have experienced from the same categories as the near-misses.

The primary predictive measures of the present study were self-reported working hours per week during the past six months. Working hours per week were grouped into the following categories: 35–40 h, 41–50 h, 51–60 h, and ≥61 h.

To assess overall sleep quality, we used the Pittsburgh Sleep Quality Index (PSQI).[20] The PSQI is an 18-item self-report questionnaire designed to assess overall sleep quality during the past month and demonstrates favorable psychometric properties. The PSQI includes the following seven components: subjective sleep quality, sleep latency, sleep duration, habitual sleep efficiency, sleep disturbances, use of sleeping medication, and daytime dysfunction. Each component score ranges from 0 to 3, with higher scores indicating greater sleep complaints. The total score ranges from 0 to 21. The cut-off point for poor sleep quality was set at 6.[20] In the present study, we used the Japanese version of the PSQI.[21]

To measure depressive symptoms, we used the Center for Epidemiological Studies Depression Scale (CES-D).[22] The CES-D is a 20-item self-rated questionnaire that measures depressive symptoms during the past week and demonstrates favorable psychometric properties. Items were rated in terms of frequency of depressive symptoms on a four-point Likert scale (0: rarely or none of the time (less than one day); 1: some or a little of the time (1–2 days); 2: occasionally or a moderate amount of time (3–4 days); and 3: most or all of the time (5–7 days)). Total scores range from 0 to 60, with higher scores indicating greater severity of depressive symptoms. Scores ≥16 suggest severe depressive symptoms.[22] In the present study, we used the Japanese version of the CES-D.[23]

To assess job-related stress, we used the Brief Job Stress Questionnaire (BJSQ),[24, 25] which has been widely used to measure job-related stress.[26] The BJSQ consists of 57 items designed to measure job stressors, including qualitative/quantitative job burden, job control, and interpersonal conflict (17 items); physical/psychological stress responses (29 items); and buffering factors, such as support from supervisors and colleagues (11 items). “High stress” was defined as meeting either of the following two criteria: (1) the highest level of stress responses or (2) moderate or higher level of stress responses along with the highest job stressors. To calculate stress response and job stressor scores, each item was rated on a four-point scale (“strongly agree,” “slightly agree,” “slightly disagree,” and “strongly disagree”). The scores for stress response and job stressors ranged from 29–116 and 26–104, respectively, with higher scores indicating higher job stressors and stress responses. The BJSQ has demonstrated acceptable or excellent reliability and validity.[24, 27] The proposed cut-off points for the “high stress” group were as follows: 77 for the stress response score for criterion (1) and 76 for the job stressor score and 63 for the stress response score for criterion (2).

As potential confounders, other demographic (sex, age, and education year) and job-related variables (e.g., presence/absence of night/shift work) were also measured.

### Statistical analysis

First, cross-tabulations were conducted between each variable and industry. To examine whether the frequency distribution of the variables, including working hours and outcomes, differed by industry, we performed chi-square tests and analysis of residuals.

To examine the association between long working hours/sleep-related problems and near-misses/injuries, multinomial logistic regression analyses were carried out with the presence/absence of injuries/near-misses as the dependent variable. The dependent variable was categorized as follows: “none (reference),” “near-miss (and no injury),” and “injury.” The logistic regression coefficients were transformed to odds ratios (ORs) with 95% confidence intervals (CIs). In the multinomial logistic regression models, we adjusted for sex, age group, education, night/shift work, job-related stress, and depression. To examine whether the association between long working hours/sleep-related problems and near-misses/injuries differed by industry, logistic regression analyses were performed by industry.

P < 0.05 was considered statistically significant. For chi-square analysis regarding the frequency of injuries and near-misses, a significance level of P < 0.01 was chosen to consider the number of significance tests undertaken. All analyses were conducted using SAS version 9.4 (SAS Institute, Cary, NC).

## Results

Of the 20,052 eligible participants, 1,007 worked less than 35 hours per week during the past six months and 363 worked less than six months of service at the time of survey; they were excluded from the statistical analyses. Data from 18,682 participants (7,098 female and 11,584 male; mean age, 43.7 [standard deviation 11.1] years) were analyzed.

The characteristics of study participants by industry are shown in Table 1. There was a significant difference in the frequency distribution for each factor by industry.

**Table 1:** Demographic, work-related, and life-related variables by industry.

Industry	Construction (n = 1,800)		Manufacturing (n = 3,888)		Transport/postal service (n = 1,289)		Wholesale/retail trade (n = 2,616)		Medical/health/welfare (n = 2,765)		Other (n = 6,324)		Total (n = 18,682)		Chi-square test
	n	(%)	n	(%)	n	(%)	n	(%)	n	(%)	n	(%)	n	(%)	
**Sex**															*
Male	1413	(78.5)	2876	(74.0)	1037	(80.4)	1433	(54.8)	712	(25.8)	4113	(65.0)	11584	(62.0)	
Female	387	(21.5)	1012	(26.0)	252	(19.6)	1183	(45.2)	2053	(74.2)	2211	(35.0)	7098	(38.0)	
**Age**															*
20–34 years	270	(15.0)	884	(22.7)	218	(16.9)	597	(22.8)	880	(31.8)	1600	(25.3)	4449	(23.8)	
35–49 years	785	(43.6)	1708	(43.9)	543	(42.1)	1216	(46.5)	1134	(41.0)	2779	(43.9)	8165	(43.7)	
50–64 years	745	(41.4)	1296	(33.3)	528	(41.0)	803	(30.7)	751	(27.2)	1945	(30.8)	6068	(32.5)	
**Education year**															*
<16 years	909	(50.5)	1624	(41.8)	759	(58.9)	1142	(43.7)	1553	(56.2)	2222	(35.1)	8209	(43.9)	
16+ years	891	(49.5)	2264	(58.2)	530	(41.1)	1474	(56.3)	1212	(43.8)	4102	(64.9)	10473	(56.1)	
**Work hours per week**															*
35–40 h	446	(24.8)	1108	(28.5)	308	(23.9)	823	(31.5)	1022	(37.0)	1932	(30.6)	5639	(30.2)	
41–50 h	715	(39.7)	1709	(44.0)	475	(36.9)	1109	(42.4)	1183	(42.8)	2495	(39.5)	7686	(41.1)	
51–60 h	340	(18.9)	679	(17.5)	235	(18.2)	395	(15.1)	313	(11.3)	1029	(16.3)	2991	(16.0)	
61+ h	299	(16.6)	392	(10.1)	271	(21.0)	289	(11.0)	247	(8.9)	868	(13.7)	2366	(12.7)	
**Night/shift work**															*
No	1458	(81.0)	3314	(85.2)	778	(60.4)	2387	(91.2)	1897	(68.6)	5177	(81.9)	15011	(80.4)	
Yes	342	(19.0)	574	(14.8)	511	(39.6)	229	(8.8)	868	(31.4)	1147	(18.1)	3671	(19.6)	
**Job-related stress**															*
Low	1423	(79.1)	3031	(78.0)	1002	(77.7)	2034	(77.8)	2018	(73.0)	4850	(76.7)	14358	(76.9)	
High	377	(20.9)	857	(22.0)	287	(22.3)	582	(22.2)	747	(27.0)	1474	(23.3)	4324	(23.1)	
**Sleep problem**															*
No	984	(54.7)	2122	(54.6)	698	(54.2)	1472	(56.3)	1424	(51.5)	3397	(53.7)	10097	(54.0)	
Yes	816	(45.3)	1766	(45.4)	591	(45.8)	1144	(43.7)	1341	(48.5)	2927	(46.3)	8585	(46.0)	
**Depression**															*
No	1150	(63.9)	2401	(61.8)	828	(64.2)	1608	(61.5)	1570	(56.8)	3857	(61.0)	11414	(61.1)	
Yes	650	(36.1)	1487	(38.2)	461	(35.8)	1008	(38.5)	1195	(43.2)	2467	(39.0)	7268	(38.9)	

* P < 0.05.

Table 2 summarizes the frequency distribution of near-misses and injuries by industry. In total, 29.1% of participants reported any type of near-miss. For all types of near-misses, a significant difference in frequency distribution was observed by industry. More than 40% of participants reported any type of near-miss in the “transport/postal services” and “medical/health/welfare” industries. Significantly more workers in the “construction” and “medical/health/welfare” industries reported “almost falling/slipping” than those in other industries. Significantly more workers in the “construction” and “transport/postal services” industries reported “almost caused traffic accident.” Significantly more workers in the “transport/postal services,” “manufacturing,” and “medical/health/welfare” industries reported “almost crashed,” “almost got caught in/between; almost got cut,” and “almost suffered other injuries,” respectively.

**Table 2:** Distribution of types of near-misses and injuries by industry.

Industry	Construction (n = 1,800)		Manufacturing (n = 3,888)		Transport/postal service (n = 1,289)		Wholesale/retail trade (n = 2,616)		Medical/health/welfare (n = 2,765)		Other (n = 6,324)		Total (n = 18,682)		Chi-square test
	n	(%)	n	(%)	n	(%)	n	(%)	n	(%)	n	(%)	n	(%)	
Any type of near-miss	572	(31.8)	1,019	(26.2)	543	(42.1)	580	(22.2)	1,228	(44.4)	1,502	(23.8)	5,444	(29.1)	*
Almost falling/slipping	272	(15.1)	410	(10.5)	167	(13.0)	197	(7.5)	445	(16.1)	498	(7.9)	1,989	(10.6)	*
Almost crashed	154	(8.6)	316	(8.1)	168	(13.0)	163	(6.2)	220	(8.0)	428	(6.8)	1,449	(7.8)	*
Almost got caught in/between; almost got cut	73	(4.1)	173	(4.4)	35	(2.7)	70	(2.7)	85	(3.1)	138	(2.2)	574	(3.1)	*
Almost caused traffic accident	223	(12.4)	262	(6.7)	294	(22.8)	220	(8.4)	238	(8.6)	456	(7.2)	1,693	(9.1)	*
Almost suffered other injuries (e.g., burn, explosion)	188	(10.4)	384	(9.9)	159	(12.3)	202	(7.7)	636	(23.0)	665	(10.5)	2,234	(12.0)	*
Any type of injuries	94	(5.2)	155	(4.0)	118	(9.2)	105	(4.0)	158	(5.7)	299	(4.7)	929	(5.0)	*
Got falling/slipping	28	(1.6)	41	(1.1)	19	(1.5)	23	(0.9)	69	(2.5)	87	(1.4)	267	(1.4)	*
Got crashed	19	(1.1)	49	(1.3)	23	(1.8)	27	(1.0)	22	(0.8)	98	(1.5)	238	(1.3)	ns
Got caught in/between; got cut	21	(1.2)	50	(1.3)	19	(1.5)	23	(0.9)	26	(0.9)	71	(1.1)	210	(1.1)	ns
Got traffic accident	32	(1.8)	23	(0.6)	58	(4.5)	34	(1.3)	21	(0.8)	69	(1.1)	237	(1.3)	*
Got other injuries (e.g., burn, explosion)	10	(0.6)	28	(0.7)	16	(1.2)	18	(0.7)	40	(1.4)	76	(1.2)	188	(1.0)	*

* P < 0.01.

Abbreviation: ns, not significant.

Regarding injuries, 5.0% of participants reported any type of injury (Table 2). Except for “got crashed” and “got caught in/between; got cut,” a significant difference in frequency distribution was observed by industry. Approximately 9% of participants reported any type of occupational injury in the “transport/postal services” industry. Significantly more workers reported “got falling/slipping” and “got other injuries” in the “medical/health/welfare” industry, while significantly more workers reported “got traffic accident” in the “transport/postal services” industry.

Table 3 shows the results of multinomial logistic regression analyses using the presence/absence of a near-miss/injuries as the dependent variable. After adjusting for sociodemographic and job-related variables, ORs for near-misses were significantly higher for those working more than 41 hours per week (41–50 h: OR = 1.2 [95% CI, 1.1–1.3]; 51–60 h: OR = 1.4 [95% CI, 1.2–1.5]; ≥61 h: OR = 1.3 [95% CI, 1.1–1.5]) than for those working 35–40 hours per week. Similarly, ORs for injuries were significantly higher for those working more than 51 hours per week (51–60 h: OR = 1.4 [95% CI, 1.1–1.8]; ≥61 h: OR = 1.5 [95% CI, 1.2–1.9]) than for those working 35–40 hours per week. Participants with sleep-related problems had significantly higher ORs for near-misses/injuries than those without these problems (near-misses: OR = 1.7 [95% CI, 1.5–1.9]; injuries: OR = 2.2 [95% CI, 1.8–2.6]).

**Table 3:** Multinomial logistic regression analysis using the presence/absence of the experience of near-misses/injuries as the dependent variable.

Category of dependent variable**	Near-miss (and no injury) (n = 4,744)		Injury (n = 929)	
	Odds ratio	(95% CI)	Odds ratio	(95% CI)
**Sex**				
Male	1.3*	(1.2–1.4)	2.3*	(1.9–2.7)
Female	1.0	(Ref)	1.0	(Ref)
**Age**				
20–34 years	1.0	(0.8–1.1)	1.5*	(1.2–1.8)
35–49 years	0.9	(0.8–1.0)	1.1	(0.8–1.3)
50–64 years	1.0	(Ref)	1.0	(Ref)
**Education year**				
<16 years	1.2*	(1.07–1.2)	1.3*	(1.1–1.6)
16+ years	1.0	(Ref)	1.0	(Ref)
**Work hours per week**				
35–40 h	1.0	(Ref)	1.0	(Ref)
41–50 h	1.2*	(1.1–1.3)	1.1	(0.8–1.3)
51–60 h	1.4*	(1.2–1.5)	1.4*	(1.1–1.8)
61+ h	1.3*	(1.1–1.5)	1.5*	(1.2–1.9)
**Night/shift work**				
No	1.0	(Ref)	1.0	(Ref)
Yes	1.7*	(1.5–1.9)	3.0*	(2.5–3.5)
**Job-related stress**				
Low	1.0	(Ref)	1.0	(Ref)
High	1.2*	(1.1–1.3)	1.0	(0.8–1.2)
**Sleep problem**				
No	1.0	(Ref)	1.0	(Ref)
Yes	1.7*	(1.5–1.9)	2.2*	(1.8–2.6)
**Depression**				
No	1.0	(Ref)	1.0	(Ref)
Yes	1.5*	(1.3–1.6)	2.7*	(2.3–3.2)

* P < 0.05.

** Reference: None.

Regarding industry (Table 4), in the “transport/postal services” (OR = 1.7 [95% CI, 1.1–2.5]) and “wholesale/retail trade” (OR = 1.5 [95% CI, 1.08–2.2]) industries, ORs for near-misses were significantly higher for those who worked more than 61 hours per week than for those who worked 35–40 hours per week. In the “transport/postal services” industry, ORs for injuries were significantly higher for those who worked more than 51 hours per week than for those who worked 35–40 hours per week (51–60 h: OR = 2.7 [95% CI, 1.3–5.6]; ≥61 h: OR = 2.7 [95% CI, 1.3–5.3]). Regardless of industry, ORs for near-misses/injuries were significantly higher for those with sleep-related problems. In almost all industries, male sex, night/shift work, and depression were associated with significantly high ORs for near-misses/injuries.

**Table 4:** Multinomial logistic regression analysis using the presence/absence of the experience of near-misses/injuries as the dependent variable by industry.

	Construction (n = 1,800)				Manufacturing (n = 3,888)				Transport/postal service (n = 1,289)			
Category of dependent variable**	Near-miss (and no injury)		Injury		Near-miss (and no injury)		Injury		Near-miss (and no injury)		Injury	
	Odds ratio	(95% CI)	Odds ratio	(95% CI)	Odds ratio	(95% CI)	Odds ratio	(95% CI)	Odds ratio	(95% CI)	Odds ratio	(95% CI)
**Sex**												
Male	3.2*	(2.2–4.5)	4.0*	(1.7–9.2)	1.5*	(1.2–1.8)	3.2*	(1.8–5.5)	1.8*	(1.2–2.6)	4.9*	(1.8–12.5)
Female	1.0	(Ref)	1.0	(Ref)	1.0	(Ref)	1.0	(Ref)	1.0	(Ref)	1.0	(Ref)
**Age**												
20–34 years	1.1	(0.7–1.5)	1.6	(0.8–2.9)	1.2	(0.9–1.5)	1.8*	(1.1–2.9)	0.8	(0.5–1.2)	0.9	(0.5–1.6)
35–49 years	0.9	(0.7–1.2)	0.9	(0.5–1.5)	0.9	(0.7–1.1)	1.2	(0.8–1.9)	0.8	(0.6–1.1)	0.6*	(0.3–0.9)
50–64 years	1.0	(Ref)	1.0	(Ref)	1.0	(Ref)	1.0	(Ref)	1.0	(Ref)	1.0	(Ref)
**Education year**												
<16 years	1.2	(0.9–1.4)	1.2	(0.7–1.9)	1.4*	(1.2–1.7)	1.3	(0.9–1.8)	1.3	(0.9–1.7)	1.0	(0.6–1.6)
16+ years	1.0	(Ref)	1.0	(Ref)	1.0	(Ref)	1.0	(Ref)	1.0	(Ref)	1.0	(Ref)
**Work hours per week**												
35–40 h	1.0	(Ref)	1.0	(Ref)	1.0	(Ref)	1.0	(Ref)	1.0	(Ref)	1.0	(Ref)
41–50 h	1.1	(0.8–1.5)	0.8	(0.4–1.5)	1.3	(1.0–1.5)	0.6	(0.4–1.0)	1.3	(0.9–1.9)	1.4	(0.7–2.8)
51–60 h	1.2	(0.8–1.7)	1.1	(0.5–2.2)	1.2	(0.9–1.6)	1.1	(0.7–1.8)	1.9*	(1.2–2.8)	2.7*	(1.3–5.6)
61+ h	1.4	(0.9–2.0)	1.5	(0.7–2.8)	1.2	(0.9–1.6)	1.0	(0.5–1.7)	1.7*	(1.1–2.5)	2.7*	(1.3–5.3)
**Night/shift work**												
No	1.0	(Ref)	1.0	(Ref)	1.0	(Ref)	1.0	(Ref)	1.0	(Ref)	1.0	(Ref)
Yes	1.4*	(1.06–1.8)	2.3*	(1.4–3.7)	1.7*	(1.3–2.1)	2.6*	(1.7–3.8)	2.2*	(1.6–2.9)	3.0*	(1.9–4.6)
**Job-related stress**												
Low	1.0	(Ref)	1.0	(Ref)	1.0	(Ref)	1.0	(Ref)	1.0	(Ref)	1.0	(Ref)
High	1.3	(0.9–1.7)	1.4	(0.8–2.3)	1.3*	(1.06–1.6)	1.0	(0.6–1.5)	1.1	(0.7–1.5)	1.0	(0.6–1.7)
**Sleep problem**												
No	1.0	(Ref)	1.0	(Ref)	1.0	(Ref)	1.0	(Ref)	1.0	(Ref)	1.0	(Ref)
Yes	1.8*	(1.4–2.3)	1.8*	(1.08–3.0)	1.9*	(1.6–2.3)	2.2*	(1.4–3.3)	2.0*	(1.5–2.7)	1.9*	(1.1–3.1)
**Depression**												
No	1.0	(Ref)	1.0	(Ref)	1.0	(Ref)	1.0	(Ref)	1.0	(Ref)	1.0	(Ref)
Yes	1.4	(1.0–1.8)	3.6*	(2.0–6.2)	1.6*	(1.3–1.9)	2.8*	(1.8–4.2)	0.9	(0.6–1.2)	1.8*	(1.09–2.9)
	Wholesale/retail trade (n = 2,616)				Medical/health/welfare (n = 2,765)				Other (n = 6,324)			
Category of dependent variable**	Near-miss (and no injury)	Injury		Near-miss (and no injury)		Injury		Near-miss (and no injury)		Injury	
	Odds ratio	(95% CI)	Odds ratio	(95% CI)	Odds ratio	(95% CI)	Odds ratio	(95% CI)	Odds ratio	(95% CI)	Odds ratio	(95% CI)
**Sex**												
Male	1.7*	(1.3–2.1)	3.2*	(1.9–5.2)	0.8	(0.6–1.0)	1.8*	(1.2–2.5)	1.1	(0.9–1.2)	1.6*	(1.1–2.2)
Female	1.0	(Ref)	1.0	(Ref)	1.0	(Ref)	1.0	(Ref)	1.0	(Ref)	1.0	(Ref)
**Age**												
20–34 years	0.9	(0.6–1.2)	2.2*	(1.1–4.2)	1.0	(0.8–1.2)	1.3	(0.8–2.0)	0.9	(0.7–1.0)	1.7*	(1.2–2.4)
35–49 years	1.0	(0.7–1.2)	2.0*	(1.1–3.6)	0.9	(0.7–1.1)	1.1	(0.6–1.7)	0.9	(0.7–1.1)	1.1	(0.8–1.5)
50–64 years	1.0	(Ref)	1.0	(Ref)	1.0	(Ref)	1.0	(Ref)	1.0	(Ref)	1.0	(Ref)
**Education year**												
<16 years	1.0	(0.8–1.3)	1.3	(0.8–1.9)	1.0	(0.8–1.2)	1.3	(0.9–1.9)	1.1	(0.9–1.2)	1.6*	(1.3–2.1)
16+ years	1.0	(Ref)	1.0	(Ref)	1.0	(Ref)	1.0	(Ref)	1.0	(Ref)	1.0	(Ref)
**Work hours per week**												
35–40 h	1.0	(Ref)	1.0	(Ref)	1.0	(Ref)	1.0	(Ref)	1.0	(Ref)	1.0	(Ref)
41–50 h	1.2	(0.9–1.5)	1.6	(0.9–2.8)	1.2*	(1.01–1.5)	1.2	(0.7–1.7)	1.2	(1.0–1.4)	1.1	(0.8–1.6)
51–60 h	1.4	(1.0–2.0)	1.2	(0.6–2.5)	1.3	(0.9–1.7)	1.3	(0.7–2.2)	1.4*	(1.1–1.7)	1.5*	(1.03–2.2)
61+ h	1.5*	(1.08–2.2)	1.9	(0.9–3.7)	1.0	(0.7–1.3)	1.3	(0.7–2.4)	1.3*	(1.07–1.6)	1.3	(0.9–1.9)
**Night/shift work**												
No	1.0	(Ref)	1.0	(Ref)	1.0	(Ref)	1.0	(Ref)	1.0	(Ref)	1.0	(Ref)
Yes	1.1	(0.7–1.5)	2.5*	(1.5–4.2)	2.1*	(1.7–2.5)	2.6*	(1.8–3.7)	1.4*	(1.2–1.7)	3.9*	(3.0–5.1)
**Job-related stress**												
Low	1.0	(Ref)	1.0	(Ref)	1.0	(Ref)	1.0	(Ref)	1.0	(Ref)	1.0	(Ref)
High	1.3	(1.0–1.7)	0.9	(0.5–1.5)	1.2	(0.9–1.4)	1.1	(0.7–1.6)	1.2	(1.0–1.4)	0.9	(0.6–1.2)
**Sleep problem**												
No	1.0	(Ref)	1.0	(Ref)	1.0	(Ref)	1.0	(Ref)	1.0	(Ref)	1.0	(Ref)
Yes	1.7*	(1.3–2.1)	2.0*	(1.2–3.1)	1.4*	(1.2–1.7)	3.5*	(2.2–5.2)	1.7*	(1.5–2.0)	2.0*	(1.5–2.7)
**Depression**												
No	1.0	(Ref)	1.0	(Ref)	1.0	(Ref)	1.0	(Ref)	1.0	(Ref)	1.0	(Ref)
Yes	1.4*	(1.1–1.8)	2.8*	(1.7–4.5)	1.3*	(1.08–1.6)	1.6*	(1.06–2.4)	1.7*	(1.4–2.0)	3.5*	(2.5–4.7)

* P < 0.05.

** Reference: None.

## Discussion

In the present study, we examined the association between long working hours/sleep-related problems and near-misses/injuries in the workplace using a nationally representative sample of workers in Japan. For all types of near-misses and some types of injuries, significant differences in frequency distributions were observed by industry. After adjustment for demographic, job-, and life-related variables, participants who worked long hours (i.e., more than 51 hours per week) were more likely to report job-related near-misses/injuries than those who worked 35–40 hours per week. The presence of sleep-related problems was also significantly related to near-misses and injuries. However, whereas the ORs for near-misses/injuries were significantly higher for those with sleep-related problems regardless of industry, the association between long working hours and near-misses/injuries differed by industry.

Nearly 30% and 5% of participants reported any types of near-misses during the past six months and injuries during the past year, respectively. Although the characteristics of the study sample and design differed from those of previous studies, the proportion of workers who experienced occupational injuries in the present study appeared to be greater than that reported in studies in developed western countries.[18] This may partially be because our nationally representative study sample included workers who were employed in industries that had the highest number of cases involving compensation for occupational injuries in Japan in 2017.[2]

For all types of near-misses and some types of injuries, a significant difference in frequency distribution was observed by industry. In particular, more than 20% of participants in the “transport/postal services” and “medical/health/welfare” industries reported “almost caused traffic accident” and “almost suffered other injuries,” respectively. Participants in the “medical/health/welfare” industry reported “almost suffered other injuries” and “got other injuries” more frequently, because this category included job-related events such as needlestick injuries, exposure to harmful substances, and musculoskeletal injuries (e.g., back pain).

After adjustment for demographic, job-, and life-related variables, participants who worked more than 51 hours per week were more likely to report job-related near-misses/injuries than those who worked 35–40 hours per week. These results are consistent with the results of previous studies on occupational injuries, suggesting that long working hours have a substantial detrimental effect on the safety of workers.[3] Our findings were also consistent with the results of previous large epidemiological studies using nationally representative samples of working adults from the United States.[8, 9] Furthermore, our findings imply a dose-response effect, in which the total number of working hours per week (over 41 hours) was positively associated with an increased risk of near-misses and injuries.

The presence of sleep-related problems was also significantly related to near-misses/injuries. Previous systematic reviews have suggested that fatigue due to long working hours, heavy workloads, and insufficient sleep/sleepiness have detrimental effects on job performance, decision-making, and, subsequently, safety.[3–6] Depending on the type of industry/occupation, workers, such as long-haul truck drivers, bus drivers, health care professionals (e.g., nurses), and flight attendants, may have irregular work schedules that could hamper sleep patterns and influence safety.[28, 29] In addition to maintaining sufficient sleep hours, screening for sleep-related problems, including obstructive sleep apnea which has been reported to be a strong risk factor for occupational injuries,[30] in industrial settings, may help prevent workplace injuries.

While sleep-related problems were significantly associated with near-misses/injuries in all industries, the association between long working hours and near-misses/injuries differed by industry. In particular, ORs for near-misses/injuries were strongly significant in the “transport/postal services” industry for those who worked more than 51 hours per week compared to those who worked 35–40 hours per week. These findings are consistent with previous studies that reported that fatigue after long periods without sleep has a negative impact on performance and accuracy for safety.[31, 32] Workers in the “transport/postal services” industry must perform their job under time pressure to meet deadlines for appointments or delivery of goods. Furthermore, we previously reported that overwork-related disorders, including *karoshi* (i.e., death by cerebrovascular and cardiovascular diseases due to overwork) and *karojisatsu* (i.e., suicide due to overwork), constitute major occupational issues in Japan, particularly among workers in the “transport/postal services” industry.[33, 34] National statistics of Japan also suggest that full-time male workers in this industry are more likely to work long hours than those engaged in other industries in Japan.[35]

Previous studies suggest that fatigue due to long working hours and poor quality of sleep are associated with medical errors and job-related injuries among workers in “medical/health/welfare” industry.[16] In contrast, the present study found that long working hours were not significantly associated with near-misses/injuries in this industry. This could be due to the fact that, as shown in Table 1, approximately 75% of participants in this industry were female. Moreover, there may be differences in the association between working hours and near-misses/injuries by occupation (i.e., physicians, nurses, or elderly care workers) in this industry. The quality and quantity of jobs may also result in differences in the association between long working hours and increased risk of near-misses/injuries.

Recent epidemiological studies regarding the safety and health of commercial truck drivers suggest that multiple rest-break periods during a trip can promote recovery from fatigue and reduce fatigue-related risk of crash/injuries.[36, 37] Periodic rest-breaks and health/fatigue management, including maintaining sufficient sleep hours may contribute to decreasing the risks of injury and overwork-related health disorders among commercial drivers. Future studies are needed to examine the detailed characteristics of near-misses and injuries in each industry, considering the differences in business practices among industries.

In addition to sleep-related problems, night/shift work was associated with significantly high ORs for near-misses/injuries in almost all industries. Irregular work patterns and night/shift work against circadian rhythm, especially nighttime driving,[28] are suggested to heighten the risk of fatigue and the risk of near-misses/injuries due to fatigue.

To our knowledge, this is the first study to examine the association between long working hours/sleep-related problems and near-misses/injuries in industrial settings using a nationally representative sample of workers. However, the current study has several limitations to note. First, we relied on self-reported job-related factors, including working hours, for exposures and near-misses/injuries as the outcome measure, which may have led to recall bias. Furthermore, in this study, the term “injury” was used to include minor injuries as well as injuries resulting in sick leave or hospitalization, and the severity of injuries remained unclear. However, previous systematic reviews on the association between long working hours and occupational injuries suggest that, regardless of the severity of injuries, working longer hours increased the risk.[3] Second, the present study was cross-sectional. Thus, causation between long working hours/sleep-related problems and near-misses/injuries cannot be determined. For example, the quality/quantity of sleep may be affected directly or indirectly through an experience of severe occupational injuries. Third, although the study sample was large and representative of the labor force population in Japan, data collection was conducted via the Internet. This may have biased the associations between long working hours/sleep-related problems and near-misses/injuries. Fourth, the present study focused on industry, rather than occupation. However, even within the same industry, a difference in frequency of near-misses/injuries may be observed by occupation, such as technical workers, clerical workers, sales/service workers, manufacturing workers, and transport/construction workers. Finally, caution should be exercised when generalizing the present findings to populations with different backgrounds, as the sample of the present study was restricted to employees in Japan.

## Conclusions

In the present study using a nationally representative sample of workers in Japan, after adjustment for sociodemographic and job-related variables, both long working hours and sleep-related problems were positively associated with safety outcomes (i.e., near-misses and injuries) among workers. However, while sleep-related problems were significantly associated with near-misses/injuries regardless of industries, the association between long working hours and near-misses/injuries differed by industry. Comprehensive protective measures for workers, including (1) reducing total hours of service/job-related fatigue, (2) maintaining sufficient sleep hours/good sleep, and (3) increasing awareness about the impact of overwork/long working hours and sleep-related problems on workers’ safety among employers, workers, clients/customers, and the general public might be effective for preventing near-misses and injuries in industrial settings among workers, especially those who work long hours in the “transport/postal services” industry.

## Supporting information

S1 Appendix Proportion of participants by industry, sex, and age group. (DOCX)

## References

[1] International Labour Organization [Internet]. Geneva: Safety and health at work [cited January 22, 2019]. Available from: https://www.ilo.org/global/topics/safety-and-health-at-work/lang—en/index.htm.

[2] Japan Industrial Safety and Health Association [Internet]. Tokyo: OSH Statistics in Japan [cited February 6, 2019]. Available from: https://www.jisha.or.jp/english/statistics/index.html.

[3] Wagstaff AS, Sigstad Lie JA. Shift and night work and long working hours: a systematic review of safety implications. Scand J Work Environ Health. 2011;37(3):173–85. 10.5271/sjweh.3146 . 21290083

[4] Robb G, Sultana S, Ameratunga S, Jackson R. A systematic review of epidemiological studies investigating risk factors for work-related road traffic crashes and injuries. Inj Prev. 2008;14(1):51–8. 10.1136/ip.2007.016766 . 18245316

[5] Kucharczyk ER, Morgan K, Hall AP. The occupational impact of sleep quality and insomnia symptoms. Sleep Med Rev. 2012;16(6):547–59. 10.1016/j.smrv.2012.01.005 . 22401983

[6] Uehli K, Mehta AJ, Miedinger D, Hug K, Schindler C, Holsboer-Trachsler E, et al Sleep problems and work injuries: a systematic review and meta-analysis. Sleep Med Rev. 2014;18(1):61–73. 10.1016/j.smrv.2013.01.004 . 23702220

[7] Smith AP. A UK survey of driving behaviour, fatigue, risk taking and road traffic accidents. BMJ Open. 2016;6(8):e011461 10.1136/bmjopen-2016-011461 27540100 PMC5013464

[8] Lombardi DA, Folkard S, Willetts JL, Smith GS. Daily sleep, weekly working hours, and risk of work-related injury: US National Health Interview Survey (2004–2008). Chronobiol Int. 2010;27(5):1013–30. 10.3109/07420528.2010.489466 . 20636213

[9] Dembe AE, Erickson JB, Delbos RG, Banks SM. The impact of overtime and long work hours on occupational injuries and illnesses: new evidence from the United States. Occup Environ Med. 2005;62(9):588–97. 10.1136/oem.2004.016667 16109814 PMC1741083

[10] Sagaspe P, Taillard J, Bayon V, Lagarde E, Moore N, Boussuge J, et al Sleepiness, near-misses and driving accidents among a representative population of French drivers. J Sleep Res. 2010;19(4):578–84. 10.1111/j.1365-2869.2009.00818.x . 20408921

[11] International Labour Organization [Internet]. ILOSTAT Database [cited February 6, 2019]. Available from: https://www.ilo.org/ilostat/faces/wcnav_defaultSelection?_afrLoop=1363940276668289&_afrWindowMode=0&_afrWindowId=null#!%40%40%3F_afrWindowId%3Dnull%26_afrLoop%3D1363940276668289%26_afrWindowMode%3D0%26_adf.ctrl-state%3Dbx2jbhsww_4.

[12] National Safety Council [Internet]. Itasca, IL: Near miss reporting systems [cited February 6, 2019]. Available from: https://www.nsc.org/Portals/0/Documents/WorkplaceTrainingDocuments/Near-Miss-Reporting-Systems.pdf.

[13] Alamgir H, Yu S, Gorman E, Ngan K, Guzman J. Near miss and minor occupational injury: does it share a common causal pathway with major injury? Am J Ind Med. 2009;52(1):69–75. 10.1002/ajim.20641 . 18942668

[14] Lander L, Eisen EA, Stentz TL, Spanjer KJ, Wendland BE, Perry MJ. Near-miss reporting system as an occupational injury preventive intervention in manufacturing. Am J Ind Med. 2011;54(1):40–8. 10.1002/ajim.20904 . 20886533

[15] Useche SA, Ortiz VG, Cendales BE. Stress-related psychosocial factors at work, fatigue, and risky driving behavior in bus rapid transport (BRT) drivers. Accid Anal Prev. 2017;104:106–14. 10.1016/j.aap.2017.04.023 . 28494258

[16] Patterson PD, Weaver MD, Frank RC, Warner CW, Martin-Gill C, Guyette FX, et al Association between poor sleep, fatigue, and safety outcomes in emergency medical services providers. Prehosp Emerg Care. 2012;16(1):86–97. 10.3109/10903127.2011.616261 22023164 PMC3228875

[17] Ministry of Internal Affairs and Communications [Internet]. Tokyo: Data set: Labour Force Survey, 2017 [cited February 6, 2019]. Available from: https://www.e-stat.go.jp/en/stat-search/files?page=1&layout=datalist&toukei=00200531&tstat=000000110001&cycle=7&year=20170&month=0&tclass1=000001040276&tclass2=000001040283&tclass3=000001040284&result_back=1.

[18] Salminen S, Oksanen T, Vahtera J, Sallinen M, Harma M, Salo P, et al Sleep disturbances as a predictor of occupational injuries among public sector workers. J Sleep Res. 2010;19(1 Pt 2):207–13. 10.1111/j.1365-2869.2009.00780.x . 19840241

[19] Ministry of Internal Affairs and Communications [Internet]. Tokyo: Japan Standard Industrial Classification, Rev. 13; c2013 [cited December 26, 2018]. Available from: http://www.soumu.go.jp/english/dgpp_ss/seido/sangyo/index.htm.

[20] Buysse DJ, Reynolds CF 3rd, Monk TH, Berman SR, Kupfer DJ. The Pittsburgh Sleep Quality Index: a new instrument for psychiatric practice and research. Psychiatry Res. 1989;28(2):193–213. . 2748771 10.1016/0165-1781(89)90047-4

[21] Doi Y, Minowa M, Uchiyama M, Okawa M, Kim K, Shibui K, et al Psychometric assessment of subjective sleep quality using the Japanese version of the Pittsburgh Sleep Quality Index (PSQI-J) in psychiatric disordered and control subjects. Psychiatry Res. 2000;97(2–3):165–72. . 11166088 10.1016/s0165-1781(00)00232-8

[22] Radloff LS. The CES-D scale: a self-report depression scale for research in the general population. Appl Psychol Meas. 1977;1:385–401. 10.1177/014662167700100306

[23] Shima S, Shikano T, Kitamura T, Asai M. Atarasii yokuutu-sei jikohyoka-syakudo ni tuite [in Japanese]. Seishin Igaku. 1985;27:717–23.

[24] Shimomitsu T, Haratani T, Ohno Y. Development of the Brief Job Stress Questionnaire mainly used for assessment of the individual: the Ministry of Labour 1999 Report. [in Japanese]. Tokyo: Tokyo Medical College; 2000.

[25] Ministry of Health, Labour and Welfare [Internet]. Tokyo: The Brief Job Stress Questionnaire English version [cited February 6, 2019]. Available from: https://www.mhlw.go.jp/bunya/roudoukijun/anzeneisei12/dl/stress-check_e.pdf.

[26] Tsutsumi A, Shimazu A, Eguchi H, Inoue A, Kawakami N. A Japanese Stress Check Program screening tool predicts employee long-term sickness absence: a prospective study. J Occup Health. 2018;60(1):55–63. 10.1539/joh.17-0161-OA 29093366 PMC5799101

[27] Tsutsumi A, Inoue A, Eguchi H. How accurately does the Brief Job Stress Questionnaire identify workers with or without potential psychological distress? J Occup Health. 2017;59(4):356–60. 10.1539/joh.17-0011-BR 28515373 PMC5557823

[28] Transportation Research Board, National Academies of Sciences, Engineering, Medicine. Commercial motor vehicle driver fatigue, long-term health, and highway safety: research needs. Washington, DC: The National Academies Press; 2016. 272 p. 27631041

[29] Institute of Medicine. Keeping patients safe: transforming the work environment of nurses. Page A, editor. Washington, DC: The National Academies Press; 2004. 484 p. 25009849

[30] Tregear S, Reston J, Schoelles K, Phillips B. Obstructive sleep apnea and risk of motor vehicle crash: systematic review and meta-analysis. J Clin Sleep Med. 2009;5(6):573–81. 20465027 PMC2792976

[31] Lamond N, Dawson D. Quantifying the performance impairment associated with fatigue. J Sleep Res. 1999;8(4):255–62. . 10646165 10.1046/j.1365-2869.1999.00167.x

[32] Williamson AM, Feyer AM. Moderate sleep deprivation produces impairments in cognitive and motor performance equivalent to legally prescribed levels of alcohol intoxication. Occup Environ Med. 2000;57(10):649–55. 10.1136/oem.57.10.649 10984335 PMC1739867

[33] Yamauchi T, Sasaki T, Yoshikawa T, Matsumoto S, Takahashi M. Incidence of overwork-related mental disorders and suicide in Japan. Occup Med (Lond). 2018;68(6):370–77. 10.1093/occmed/kqy080 . 29897506

[34] Yamauchi T, Yoshikawa T, Takamoto M, Sasaki T, Matsumoto S, Kayashima K, et al Overwork-related disorders in Japan: recent trends and development of a national policy to promote preventive measures. Ind Health. 2017;55(3):293–302. 10.2486/indhealth.2016-0198 28154338 PMC5462645

[35] Ministry of Health, Labour and Welfare [Internet]. Tokyo: 2016 White paper on preventive measures against overwork-related disorders (Karoshi tou boushi taisaku hakusyo) [in Japanese] [cited February 6, 2019]. Available from: http://www.mhlw.go.jp/wp/hakusyo/karoushi/16/index.html.

[36] Chen GX, Sieber WK, Lincoln JE, Birdsey J, Hitchcock EM, Nakata A, et al NIOSH national survey of long-haul truck drivers: injury and safety. Accid Anal Prev. 2015;85:66–72. 10.1016/j.aap.2015.09.001 26397196 PMC4631642

[37] Chen C, Xie Y. The impacts of multiple rest-break periods on commercial truck driver's crash risk. J Safety Res. 2014;48:87–93. 10.1016/j.jsr.2013.12.003 . 24529096

