# Supplementary material for: Long working hours, sleep-related problems, and near-misses/injuries in industrial settings using a nationally representative sample of workers in Japan
Source: PLoS One. 2019 Jul 15;14(7):e0219657. doi: 10.1371/journal.pone.0219657 (PMC6629083; doi:10.1371/journal.pone.0219657)
Supplement: S1 Appendix — (DOCX) [file pone.0219657.s001.docx]

**S1 Appendix. Proportion of participants by industry, sex, and age group**

|  |  | Present study | | | | | | | | |  | Labour Force Survey, 2017 | | | | | | | | |
| --- | --- | --- | --- | --- | --- | --- | --- | --- | --- | --- | --- | --- | --- | --- | --- | --- | --- | --- | --- | --- |
|  |  | Men | | |  | Women | | |  | Total |  | Men | | |  | Women | | |  | Total |
|  | Age group Industry | 20-34 | 35-49 | 50-64 |  | 20-34 | 35-49 | 50-64 |  | 20-64 |  | 20-34 | 35-49 | 50-64 |  | 20-34 | 35-49 | 50-64 |  | 20-64 |
| 1 | Agriculture/forestry | 2% | 2% | 3% |  | 1% | 2% | 1% |  | 2% |  | 2% | 2% | 3% |  | 1% | 1% | 3% |  | 2% |
| 2 | Fishing | 0% | 0% | 0% |  | 0% | 0% | 0% |  | 0% |  | 0% | 0% | 0% |  | 0% | 0% | 0% |  | 0% |
| 3 | Mining | 0% | 0% | 0% |  | 0% | 0% | 0% |  | 0% |  | 0% | 0% | 0% |  | 0% | 0% | 0% |  | 0% |
| 4 | Construction | 8% | 12% | 13% |  | 3% | 5% | 6% |  | 8% |  | 9% | 12% | 13% |  | 2% | 3% | 3% |  | 8% |
| 5 | Manufacturing | 24% | 22% | 19% |  | 12% | 13% | 12% |  | 17% |  | 22% | 22% | 20% |  | 10% | 12% | 11% |  | 17% |
| 6 | Electricity/gas/heating/water | 1% | 1% | 1% |  | 0% | 0% | 0% |  | 0% |  | 0% | 1% | 1% |  | 0% | 0% | 0% |  | 0% |
| 7 | Information/communication | 5% | 5% | 2% |  | 3% | 2% | 1% |  | 3% |  | 5% | 6% | 3% |  | 3% | 2% | 1% |  | 4% |
| 8 | Transport/postal services | 7% | 8% | 9% |  | 3% | 4% | 3% |  | 6% |  | 5% | 8% | 9% |  | 2% | 3% | 2% |  | 5% |
| 9 | Wholesale/retail trade | 11% | 14% | 13% |  | 18% | 17% | 17% |  | 15% |  | 15% | 14% | 13% |  | 20% | 19% | 20% |  | 16% |
| 10 | Finance/insurance | 2% | 2% | 2% |  | 3% | 3% | 3% |  | 2% |  | 2% | 2% | 3% |  | 4% | 3% | 3% |  | 3% |
| 11 | Real estate/leasing | 2% | 2% | 3% |  | 1% | 1% | 2% |  | 2% |  | 2% | 2% | 2% |  | 1% | 2% | 2% |  | 2% |
| 12 | Scientific/technical services | 2% | 4% | 4% |  | 2% | 3% | 2% |  | 3% |  | 3% | 4% | 4% |  | 3% | 3% | 2% |  | 4% |
| 13 | Accommodation/food services | 4% | 3% | 3% |  | 8% | 6% | 5% |  | 4% |  | 6% | 3% | 3% |  | 9% | 7% | 7% |  | 5% |
| 14 | Entertainment | 1% | 2% | 2% |  | 4% | 3% | 3% |  | 2% |  | 3% | 2% | 2% |  | 5% | 4% | 4% |  | 3% |
| 15 | Education/learning support | 3% | 3% | 4% |  | 6% | 5% | 7% |  | 5% |  | 4% | 3% | 4% |  | 7% | 6% | 7% |  | 5% |
| 16 | Medical/health/welfare | 8% | 5% | 5% |  | 24% | 23% | 22% |  | 13% |  | 7% | 5% | 5% |  | 23% | 23% | 22% |  | 13% |
| 17 | Compound services | 1% | 1% | 1% |  | 1% | 1% | 1% |  | 1% |  | 1% | 1% | 1% |  | 1% | 1% | 1% |  | 1% |
| 18 | Other services | 5% | 5% | 8% |  | 3% | 5% | 8% |  | 6% |  | 5% | 6% | 7% |  | 5% | 5% | 7% |  | 6% |
| 19 | Public service | 5% | 5% | 3% |  | 2% | 2% | 2% |  | 3% |  | 6% | 5% | 5% |  | 3% | 3% | 2% |  | 4% |
| 20 | Other | 8% | 5% | 4% |  | 6% | 5% | 5% |  | 5% |  | 2% | 1% | 1% |  | 2% | 2% | 1% |  | 2% |
|  | Total | 100% | 100% | 100% |  | 100% | 100% | 100% |  | 100% |  | 100% | 100% | 100% |  | 100% | 100% | 100% |  | 100% |
